# Racial and Ethnic Disparities in Low-Risk Unplanned Cesarean Birth: Disaggregating Asian Data

**DOI:** 10.1007/s40615-025-02401-0

**Published:** 2025-03-26

**Authors:** Sydney M. Spencer, Amy A. Laurent, Vivienne L. Souter, Ian S. Painter, Colleen M. Daly

**Affiliations:** 1 Microsoft Corporation, Redmond, WA USA; 2 Department of Health Systems & Population Health, University of Washington, Seattle, WA USA; 3 Foundation for Health Care Quality, Seattle, WA USA

**Keywords:** Asian, Cesarean, Disaggregation, Ethnicity, Health disparities, NTSV

## Abstract

**Background:**

Cesarean outcomes are rarely investigated by Asian ethnicities when examining variation among low-risk, first-time birthing parents. We analyzed a clinical birth dataset of Northwestern U.S. hospitals to evaluate disparities in unplanned cesarean births among disaggregated Asian ethnicities.

**Methods:**

This cross-sectional study used chart-abstracted birth data from 2017 through 2021. Analysis restrictions included hospitals reporting for the full timeframe, and patients who were nulliparous, term, singleton, vertex presentation, allowed to labor without a scheduled cesarean birth, and not intrapartum transfers or community births. Adjusted and unadjusted multi-level logistic regression compared the primary outcome of unplanned cesarean birth by race and Asian ethnicities.

**Results:**

A total of 40,160 births met inclusion criteria; 21.3% were Asian. Overall, the laboring cesarean rate was 23.1%, ranging from 33.9% for South Asians to 17.0% for East Asians. Compared to Whites, South Asians (OR 1.84, CI 1.66–2.04), Southeast Asians (OR 1.28, CI 1.05–1.55), and Asian unspecified (OR 1.27, CI 1.18–1.37) had significantly higher unadjusted odds of cesarean birth while East Asians had significantly lower odds (OR 0.73, CI 0.63–0.86). Odds for South Asian cesarean birth were more than doubled that of White births (aOR 2.18, CI 1.95–2.44) in the adjusted model.

**Conclusions:**

After controlling for known risk factors, South Asians had elevated odds for unplanned cesarean birth compared to other races and ethnicities, despite lower risk factor incidence. Medical systems should collect disaggregated race and ethnicity data to provide pregnancy management insights for reducing inequities in low-risk unplanned cesarean births.

**Supplementary Information:**

The online version contains supplementary material available at 10.1007/s40615-025-02401-0.

**Classification code:** MSC: 62J12, 97K40.

## Background

Despite initiatives for reducing cesarean births, rates in the USA remain high, especially in nulliparous, term, singleton, vertex presentation (NTSV) births, which are classified as low risk.In 2021, the national cesarean rate for first time births was 26.3%, well above the 23.6% Healthy People 2030 goal [1]. Cesarean births are associated with increased maternal morbidity and mortality [2] and contribute to increased healthcare costs [3].

Risk factors for cesarean birth are well documented in the population as a whole, [4, 5] and literature on racial and ethnic differences show disparities across birthing parents of color [5–7]. However, these studies primarily use the 1997 Office of Management and Budget (OMB) race categories (American Indian/Alaska Native, Asian, Black/African American, Hispanic, Native Hawaiian/Pacific Islander, White, and multiracial) or the 1977 OMB racial groups that collapse Asian and Pacific Islander; many of the studies do not examine data by all seven of the OMB categories[8]. While improved, even the expanded 1997 OMB categorization still encapsulates very heterogenous populations with different risk factors and health outcomes [9, 10], particularly for individuals of Asian ethnicity. Previous research has called for disaggregating the Asian group to explore evidence of health disparities by subethnicities [11]. Disease heterogeneity in disaggregated Asian groups is likely multifactorial, including cultural, lifestyle, socioeconomic, and genetic differences between subgroups, as well as influences from early life and environmental exposures and health care access, particularly in immigrant populations [12]. Additionally, individuals of Asian ethnicity in the USA differ significantly by income, age, education, and other characteristics; this highlights the wide diversity of the US Asian population [13]. Limited research on Asian pregnancy outcomes in the US shows differences in preterm labor, primary cesarean delivery, low birth weight, macrosomia, and cephalopelvic disproportion [14]. However, a lack of disaggregated data on Asian groups in most clinical datasets has been a barrier to more extensive research.

Understanding disparities for cesarean birth for the Asian population is especially relevant in Washington state where Asian residents made up the largest and fastest growing racial minority group in 2020 (10.3%) [15, 16]. Compared to their representation in overall state racial demographics, Asian residents are overrepresented in the local Washington state technology workforce. At large Washington-based technology companies, Asian employees comprise between 15 and 37% of the workforce [17–19]. Employers are purchasers of healthcare coverage for nearly half of Americans, making them well-positioned to impact healthcare delivery [20]. Therefore, understanding cesarean disparities by racial and ethnic demographics is imperative for informing equitable health plan design.

Our objective was to use a granular, contemporary clinical dataset that includes information on self-reported Asian groups, to evaluate odds of and risk factors for unplanned cesarean NTSV births by disaggregated Asian ethnic groups compared to other racial and ethnic groups in the Northwestern US.

## Methods

This cross-sectional study used data from the Foundation for Health Care Quality’s (FHCQ) Obstetrical Care Outcomes Assessment Program (OB COAP), a multicenter, continuous perinatal quality improvement initiative started in Washington state. The OB COAP dataset captures chart-abstracted clinical variables from medical records uploaded and entered by trained abstractors from births of 20 weeks’ gestation or greater and does not rely on administrative billing data. Fields include maternal demographics, pre-pregnancy health, pregnancy complications, labor and delivery course, and postnatal outcomes for birthing persons and newborns; the dataset has been fully described elsewhere [21]. The de-identified and coded data used in this study were collected for purposes of quality improvement. The Washington State Institutional Review Board (WSIRB) has deemed research using de-identified OB COAP data as non-human subjects research and thereby exempt from IRB review and patient consent.

The study population included nulliparous, term (37^+0^–42^+0^ weeks’ gestation) singleton vertex births (NTSV) at 14 hospitals based in the Northwestern US—a majority of which are located in Washington state, that reported continuously to OB COAP between January 2017 and December 2021. Intrapartum transfers to and from the hospital, planned community births, and cesareans performed without a trial of labor were excluded.

The outcome of interest for this analysis was unplanned cesarean birth occurring after a trial of labor. The exposure was self-reported race and ethnicity as recorded by the hospital in the medical record captured in the OB COAP database. Mutually exclusive broad race and ethnic groups analyzed were American Indian/Alaskan Native, Asian/Asian American, Black/African American, Hispanic/Latinx, Native Hawaiian/Pacific Islander, missing/unspecified, multiracial (patient selected more than one non-Hispanic/Latinx categories or selected the multiracial group), other, and White. The OB COAP dataset captures the following four Asian ethnic groups: Asian unspecified, East Asian (Chinese, Japanese, Korean), Southeast Asian (Cambodian, Laotian, Malaysian, Filipino, Thai, Vietnamese), and South Asian (Indian, Pakistan, Nepalese, Bhutanese, Sri Lankan, Maldivian). These groups were used for the descriptive analysis of population characteristics and the multivariable analyses. If the composition of the Asian unspecified group matched the distribution in Washington state, the majority would be East Asian, followed by Southeast and South Asian [13]. However, since we could not validate this, we analyzed the unknown group as a separate category.

To understand how cesarean birth differed by race and ethnicity, unadjusted and adjusted odds ratios were calculated using a multi-level multivariable mixed-effect logistic regression model. Covariates included variables available in the OB COAP dataset that had previously been identified in published literature as risk factors for cesarean birth [22].

The model included the following independent variables: age at delivery, year of delivery, final body mass index (< 18.5, 18.5–24.9, 25–29.9, 30–34.9, 35–39.9, or ≥ 40) either upon admission or at the last prenatal visit before birth, baby weight in grams at birth (< 2500 g, 2500–4000 g, or > 4000 g), insurance status (commercial or government/other), pre-pregnancy diagnosis of hypertension, pre-pregnancy diagnosis of diabetes, pre-eclampsia/gestational hypertension, gestational diabetes, induction of labor, hospital neonatal level of care [23], absent/minimal prenatal care (assessed by provider), prenatal provider type and admission provider type (family practice, midwife, obstetrician, maternal fetal medicine specialist, or other), and the hospital of birth. Macrosomia was defined as birthweight over 4000 g and low birthweight was under 2500 g. The Economic Innovation Group’s Distressed Community Index (DCI) was applied at the ZIP code level of the patient’s residence and included as a covariate. The DCI provides aerial-based quintiles approximating socioeconomic status; areas with a quintile of 1 are the most affluent and quintile 5 are the most distressed [24, 25]. Birthing parents’ gender was not captured. Missing or implausible values for covariate variables were coded as separate levels and included in the analysis. Hospital of birth was included as a random intercept.

Finally, to investigate potential causes for racial and ethnic variation in cesarean rates, we examined indications for cesarean by race and ethnicity. We calculated unadjusted and adjusted odds ratios for cesarean indications by race and ethnicity using multi-level multivariable logistic regression (*p* < 0.05). Covariates for the model were the same as those included in the model examining differences unplanned cesarean birth by race and ethnicity (described above). We used the largest of the groups, the White population, as the reference group throughout. All analyses were conducted using Stata version 17.0 [26].

## Results

**Fig. 1:**
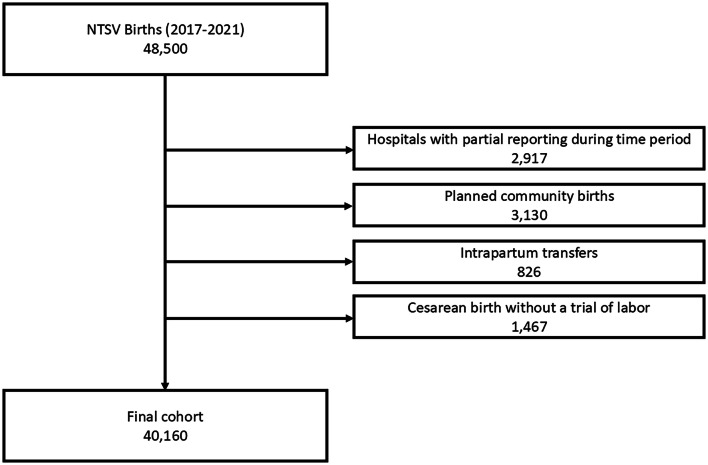
Study population

Between January 2017 and December 2021, 48,500 NTSV births were captured in the dataset. A total of 40,160 births met the inclusion criteria (Fig. 1). Of these, 21.3% occurred in Asian birthing parents (12.4% Asian unspecified, 4.6% South Asian, 2.9% East Asian, and 1.4% Southeast Asian; Table 1). Data on race and ethnicity were missing in 5.6% of births. Rates of cesarean risk factors for the non-Asian racial groups were similar to nationally reported disparities [27]. This article focused on results for Asian births because disaggregated ethnicities have been underexplored within maternal and birthing health disparities research [27]. More data on other racial groups can be found in Table 1 in the Supplementary Information. The mean age at birth was 28.4 (SD 5.51) for all births, 30.7 (SD 4.12) for Asian births, 30.6 (4.26) for Asian unspecified births, 31.5 (SD 3.84) for East Asian births, 30.4 (SD 4.80) for Southeast Asian births, and 30.2 (SD 3.60) for South Asian births (Table 1).

**Table 1:** Patient demographics by Asian ethnic groups

**Factor**		Asian **ethnic groups**				Total **study population**
	*Asian (total)	**Asian unspecified**	**East Asian**	**Southeast Asian**	**South Asian**	
**Age** **Mean (SD)**	30.7 (4.12)	30.6 (4.26)	31.5 (3.84)	30.4 (4.80)	30.2 (3.60)	28.4 (5.52)
**BMI at final prenatal visit** **Median (SD)**	28.0 (25.5, 30.9)	28.0 (25.5, 31.0)	25.9 (24.1, 28.4)	28.2 (25.8, 31.3)	29.0 (26.7, 31.6)	30.3 (27.1, 34.6)
**Baby weight (%)**						
< 2500 g	348 (4.1)	203 (4.1)	32 (2.8)	21 (3.8)	92 (5.0)	1059 (2.6)
2500–4000 g	7878 (92.2)	4577 (91.7)	1091 (94.4)	512 (93.4)	1698 (91.7)	35,979 (89.6)
> 4000 g	320 (3.7)	211 (4.2)	33 (2.9)	15 (2.7)	61 (3.3)	3122 (7.8)
**Insurance type (%)**						
Government/other	911 (10.7)	586 (11.7)	112 (9.7)	107 (19.5)	106 (5.7)	11,792 (29.4)
Commercial	7557 (88.4)	4364 (87.4)	1009 (87.3)	440 (80.3)	1744 (94.2)	28,046 (69.8)
Missing/unspecified	78 (0.9)	41 (0.8)	35 (3.0)	1 (0.2)	1 (0.1)	322 (0.8)
**Pre-pregnancy hypertension (%)**	123 (1.4)	86 (1.7)	2 (0.2)	10 (1.8)	25 (1.4)	1039 (2.6)
**Pre-pregnancy diabetes (%)**	123 (1.4)	77 (1.5)	11 (1.0)	9 (1.6)	26 (1.4)	458 (1.1)
**DCI quintile (%)**						
1 (least distressed)	5786 (67.7)	3247 (65.1)	890 (77.0)	271 (49.5)	1378 (74.4)	17,719 (44.1)
2	1742 (20.4)	1042 (20.9)	195 (16.9)	138 (25.2)	367 (19.8)	10,574 (26.3)
3	668 (7.8)	433 (8.7)	47 (4.1)	100 (18.2)	88 (4.8)	4804 (12.0)
4	262 (3.1)	200 (4.0)	13 (1.1)	35 (6.4)	14 (0.8)	5067 (12.6)
5 (most distressed)	51 (0.6)	43 (0.9)	6 (0.5)	2 (0.4)	0 (0.0)	1577 (3.9)
Missing	37 (0.4)	26 (0.5)	5 (0.4)	2 (0.4)	4 (0.2)	419 (1.0)
**Preeclampsia/gestational hypertension (%)**	745 (8.7)	462 (9.3)	65 (5.6)	64 (11.7)	154 (8.3)	5587 (13.9)
**Gestational diabetes (%)**	1457 (17.0)	806 (16.1)	206 (17.8)	107 (19.5)	338 (18.3)	3798 (9.5)
**Induced (%)**	3219 (37.7)	1821 (36.5)	370 (32.0)	204 (37.2)	824 (44.5)	16,817 (41.9)
**Neonatal level of care** (%**)**						
1	105 (1.2)	65 (1.3)	13 (1.1)	14 (2.6)	13 (0.7)	2752 (6.8)
2	658 (7.7)	468 (9.4)	84 (7.3)	47 (8.6)	59 (3.2)	8514 (21.2)
3 and 4	7783 (91.1)	4458 (89.3)	1059 (91.6)	487 (88.9)	1779 (96.1)	28,894 (72.0)
**Absent/minimal prenatal care (%)**	96 (1.1)	60 (1.2)	5 (0.4)	20 (3.6)	11 (0.6)	1096 (2.7)

*Numerator includes Asian-Unspecified, East Asian, Southeast Asian, and South Asian, denominator encompasses total study population

**Births by prenatal provider type, admission provider type and hospital not shown (covariates included in multivariable model)

*n *= 40,160

Considering socioeconomic indicators, the proportion of commercially insured births was greater for Asian birthing parents compared to the total population (88.4% versus 69.8% respectively). Within the Asian ethnicities, Southeast Asian births had the lowest proportion of commercially insured births (80.3%) in this group while South Asian births had the highest proportion (94.2%) (Table 1). Asian births had the greatest proportion of *least* economic distress (DCI score of 1 out of 5) [24] compared to all other race categories (67.7% versus 44.1% for total births). Proportions for the *least* distressed score varied by Asian ethnic group: 77.0% for East Asian births, 74.4% for South Asian births, 65.1% for Asian unspecified births, and 49.5% for Southeast Asian births.

Regarding unplanned cesarean risk factors, the proportion of gestational diabetes was higher for Asian births (17.0%) compared to total births (9.5%). Southeast Asian births had the highest proportion of gestational diabetes (19.5%), followed by South Asian births (18.3%), East Asian births (17.8%), and Asian unspecified (16.1%) (Table 1). Hypertension was lower for Asian births (8.7%) compared to total births (13.9%) and, by Asian ethnicity, ranged from 5.6% for East Asian births to 11.7% for Southeast Asian births. Asian births had smaller proportions of macrosomia (3.7%) compared to the total population (7.8%) ranging from 2.7% for Southeast Asian births to 4.2% for Asian unspecified. Total induction rates for the population were 41.9% but ranged from 32.0% for East Asian births to 44.5% for South Asian births. Additional risk factors can be found in Table 1.

Unplanned cesarean rate, the primary outcome, was 23.1% for total births, similar to national targets for cesarean NTSV births [1]. However, wide variations in unplanned cesarean birth among Asian ethnic groups were observed. Exceeding the national target, South Asians (33.9%) had the highest rate, followed by Southeast Asians (26.3%) and Asian unspecified (26.3%), while East Asians were well below the national target (17.0%) (Table 2).

**Table 2:** Adjusted and unadjusted odds for unplanned cesarean birth

	**Total** **(%)**	**Unplanned cesarean (%)**	**OR** **(95% CI)**	**aOR** **(95% CI)**
**Broad race categories^**				
AI/AN	403 (1.0)	87 (21.6)	0.99 (0.78,1.26)	1.22 (0.94,1.59)
Asian (total)	8546 (21.3)	2273 (26.6)	*1.30 (1.23,1.38)	*1.65 (1.54,1.77)
B/AA	1916 (4.8)	587 (29.3)	*1.59 (1.44,1.76)	*1.91 (1.70,2.14)
H/L	4916 (12.2)	977 (19.9)	*0.89 (0.82,0.96)	*1.25 (1.13,1.36)
NH/PI	501 (1.3)	127 (21.1)	*1.39 (1.14,1.70)	*1.49 (1.20,1.85)
Multiracial	603 (1.5)	140 (27.9)	0.96 (0.79,1.17)	1.18 (0.95,1.46)
Other	908 (2.3)	191 (21.0)	0.96 (0.81,1.13)	*1.22 (1.02,1.45)
White	20,132 (50.1)	4389 (21.8)	–	–
Missing	2235 (5.6)	499 (22.3)	1.03 (0.93,1.15)	*1.15 (1.02, 1.30)
**Asian ethnic groups**				
Asian-UNS	4991 (12.4)	1306 (26.2)	*1.27 (1.18,1.37)	*1.60 (1.48, 1.73)
East Asian	1,156 (2.9)	196 (17.0)	*0.73 (0.63,0.86)	1.06 (0.90,1.26)
Southeast Asian	548 (1.4)	144 (26.3)	*1.28 (1.05,1.55)	*1.75 (1.42, 2.15)
South Asian	1851 (4.6)	627 (33.9)	*1.84 (1.66,2.04)	*2.18 (1.95, 2.44)
**Total**	40,160 (100.0)	9,272 (23.1)		

*p < 0.05

- reference group

^*AI/AN*, American Indian/Alaska Native; *B/AA*, Black/African America; *H/L,* Hispanic/Latinx; *NH/PI,* Native Hawaiian/Pacific Islander; *UNS*, Unspecified, *n *= 40,160

In aggregate, Asian births had significantly greater odds for unplanned cesarean births compared to White births (OR 1.30, 95% CI 1.23, 1.38). Differences in odds of unplanned cesarean birth were observed among Asian ethnic groups. Compared to White births, South Asian (OR 1.84, 95% CI 1.66, 2.04), Southeast Asian (OR 1.28, 95% CI 1.05, 1.55), and Asian unspecified births (OR 1.27, 95% CI 1.18, 1.37) had significantly higher odds while East Asian births had significantly lower odds (OR 0.73, 95% CI 0.63, 0.86) (Table 2).

In the adjusted model, aggregated Asian births had significantly higher odds for unplanned cesarean compared to White births (aOR 1.65, 95% CI 1.54, 1.77). After adjustment, South Asian births had the highest odds for unplanned cesarean (aOR 2.18, 95% CI 1.95, 2.44) compared to White births followed by Southeast Asian (aOR 1.75, 95% CI 1.42, 2.15) and Asian unspecified (aOR1.60, 95% CI 1.48, 1.72). Odds for the East Asian group were not significant in the adjusted model when compared to White births (Table 2).

In the sub-analysis exploring indications for unplanned cesarean births, 59.9% of births had slow/no progress in labor, 34.9% had concern for fetal condition, and 5.2% fell in the other category (Table 3). In the unadjusted analysis, Southeast Asian births had significantly lower odds for concern for fetal condition as an indication for cesarean (OR 0.62, 95% CI 0.38, 0.84) and significantly higher odds of slow/no progress in labor as an indication (OR 1.56, 95% CI 1.08, 2.25) compared to White births. The results for Southeast Asian births maintained significance in the adjusted analysis with this group having lower odds of concern for fetal condition (aOR 0.56, 95% CI 0.28, 0.84) and higher odds for slow/no progress in labor (aOR 1.77, 95% CI 1.21, 2.57) compared to White births. South Asian births, compared to White births, had significantly higher odds for concern for fetal condition (OR 1.25, 95% CI 1.05, 1.49) although results were not significant in the adjusted analysis. Odds were not significant for the Asian total births and all other Asian ethnic groups.

**Table 3:** Indications for cesarean in the unplanned cesarean NTSV population

	**Concern for fetal condition**			**Slow/no progress in labor**			**Other indication**		
	**Total (%)**	**OR**	**aOR**	**Total (%)**	**OR**	**aOR**	**Total (%)**	**OR**	**aOR**
**Broad racial groups**									
AI/AN	31 (35.6)	1.13 (0.73,1.35)	1.10 (0.69,1.75)	50 (57.5)	0.86 (0.56,1.33)	0.87 (0.55,1.37)	5 (5.8)	1.15 (0.46,2.88)	1.26 (0.49,3.23)
Asian (total)	802 (35.3)	1.10 (0.99,1.23)	0.96 (0.85,1.09)	1353 (59.5)	0.92 (0.83,1.02)	1.05 (0.93,1.18)	109 (4.8)	0.95 (0.75,1.20)	0.95 (0.73,1.23)
B/AA	267 (45.3)	*1.70 (1.43,2.03)	*1.36 (1.13,1.64)	292 (49.6)	*0.62 (0.52,0.74)	*0.77 (0.64,0.93)	24 (4.1)	0.80 (0.52,1.23)	0.80 (0.51,1.26)
H/L	324 (33.2)	1.00 (0.87,1.16)	*0.83 (0.70,0.97)	583 (59.7)	0.93 (0.80,1.07)	1.09 (0.93,1.28)	66 (6.8)	*1.36 (1.02,1.81)	*1.45 (1.06, 1.98)
NH/PI	57 (40.7)	1.40 (0.99,1.97)	1.27 (0.89,1.82)	74 (52.9)	*0.71 (0.50,0.99)	0.79 (0.55,1.12)	8 (5.7)	1.14 (0.55,2.36)	1.08 (0.51,2.27)
Multiracial	40 (31.5)	0.93 (0.63,1.35)	0.78 (0.52,1.15)	82 (64.6)	1.13 (0.79, 1.44)	1.35 (0.92,1.98)	5 (3.9)	0.77 (0.31,1.89)	0.74 (0.30,1.87)
Other	60 (31.4)	0.93 (0.68,1.27)	0.81 (0.58,1.11)	120 (62.8)	1.06 (0.78,1.63)	1.23 (0.90,1.67)	10 (5.2)	1.04 (0.54,1.99)	0.97 (0.50,1.90)
White	1451 (33.1)	-	-	2699 (61.5)	-	-	222 (5.0)	-	-
Missing	192 (38.5)	*1.28 (1.05,1.54)	1.12 (0.90,1.38)	277 (55.5)	0.79 (0.65,0.95)	0.91 (0.74,1.12)	26 (5.2)	1.04 (0.68,1.57)	0.95 (0.60,1.49)
**Asian ethnic groups**									
Asian-UNS	463 (35.4)	1.12 (0.98,1.27)	0.96 (0.84,1.11)	774 (59.3)	0.92 (0.81, 1.04)	1.05 (0.91,1.20)	61 (4.7)	0.92 (0.69,1.23)	0.94 (0.69,1.28)
East Asian	65 (33.2)	1.00 (0.74,1.35)	0.85 (0.62,1.17)	121 (61.7)	1.00 (0.74,1.34)	1.17 (0.86,1.59)	10 (5.1)	1.01 (0.52,1.93)	0.98 (0.65,1.49)
SE Asian	34 (23.6)	*0.62 (0.42,0.92)	*0.56 (0.38,0.84)	103 (71.5)	*1.56 (1.08,2.25)	*1.77 (1.21,2.57)	7 (4.9)	0.96 (0.44,2.07)	0.86 (0.39,1.88)
South Asian	240 (38.3)	*1.25 (1.05,1.49)	1.14 (0.94,1.38)	355 (56.6)	*0.81 (0.69,0.96)	0.89 (0.74,1.07)	31 (4.9)	0.97 (0.66,1.43)	0.98 (0.65,1.49)
**Total**	3224 (34.9)			5530 (59.9)			475 (5.2)		

*p < 0.05

- reference group

*AI/AN*, American Indian/Alaska Native; *B/AA*, Black/African America; *H/L*, Hispanic/Latinx; *NH/PI,* Native Hawaiian/Pacific Islander; *UNS,* Unspecified; *SE*, Southeast

## Discussion

Using clinical data from the Northwestern US, our study filled an important gap on birthing disparities within Asian ethnicities — a population that is frequently omitted from sexual and reproductive health research [27]. Few studies explored disparities in unplanned cesarean birth by disaggregated Asian ethnicities, even when there was a specific focus on reporting Asian birth outcomes [5, 28, 29]. Our data showed Asian births have increased odds for an unplanned cesarean in both adjusted (aOR1.65, 95% CI 1.54,1.77) and non-adjusted (OR 1.30, 95% CI 1.23,1.38) analyses in comparison to White births. When disaggregating the Asian racial group, our study found South Asian, Southeast Asian, and Asian unspecified births had increased odds for unplanned cesarean compared to White births in the unadjusted and adjusted analyses. East Asian births had significantly lower odds of unplanned cesarean compared to White births although this did not remain significant in the adjusted analyses**.**

Composition of Asian ethnic groups in the USA may vary by region, limiting the generalizability of our findings. However, three studies exploring cesarean disparities have also observed variation by disaggregated Asian subgroups, eluding to consistent patterns of heterogeneity within the Asian race group [30–32]. In a populous state of the Northeastern US, East Asian (Japanese, Chinese, and Korean) singleton, term, and vertex births had significantly lower odds of primary cesarean while Asian Indian, Filipino, and other Asian groups had significantly higher odds compared to White births [30]. In Massachusetts, NTSV births that identified as Asian Indian and Filipino had significantly higher odds of primary cesarean while Japanese, Chinese, and Cambodian had significantly lower odds compared to non-Hispanic, non-black births [31]. In New York City, South Asian singleton births had significantly greater risk for cesarean while East Asian births had significantly lower risk compared to non-Hispanic White births [32].

In our study, South Asians had the highest odds for unplanned cesarean birth compared to White births despite living in areas of higher socioeconomic status and having lower rates of pre-eclampsia/hypertension, factors often associated with reduced risk for adverse birthing outcomes [33]. The South Asian population had the second highest rate of GDM, a commonly cited risk factor for unplanned cesarean birth. However, South Asian odds for unplanned cesarean birth remained high compared to Whites births despite controlling for GDM. There is scant literature examining risk for unplanned cesarean birth in the South Asian population. One previous study found increased odds for unplanned cesarean in South Asian births compared to White births, but the analysis was not restricted to the NTSV population [32]. Another study exploring potential risk factors for unplanned cesarean in Asian Indian nulliparous, term, and singleton births observed risk for the outcome significantly increased at a baby weight of 3000 g compared to White births, hypothesizing that Asian Indian birthing parents in the USA have larger babies than in India [34], citing a World Health Organization report of a 2975-g birthweight average in India [35] compared to 3143 g in a study of 10 million US births [36].

Clinically, indication for cesarean is a metric that has been used to understand why birthing parents received a cesarean. In our data, we did not see obvious reasons explaining these differences in unplanned cesarean birth. Southeast Asian births were significantly more likely compared to White births to have slow/no progress of labor as an indication for cesarean and significantly less likely to have concern for fetal condition as an indication. No significant odds for a specific cesarean indication category were observed for the other Asian groups in the adjusted model.

One driver of high odds for unplanned cesarean birth in the South Asian population could be cultural discordance between medical providers and patients. Cultural discordance has been previously suggested as a potential explanation for increased cesarean rates for other racially minoritized patients [37]. A qualitative study of provider and medical staff non-medical explanations for high cesarean rates in Micronesian Hawaiian birthing parents reported problems with communication, interpretation, family involvement, and negative attitudes and stereotypes as meaningful factors [38]. The OB COAP dataset did not allow for an analysis around provider and patient racial concordance, but nonmedical factors could have potentially played a role.

Provider bias may be a contributor to higher risk of unplanned cesarean birth for patients. Perceived pressures from a provider to have a cesarean as well as labor management practices have been shown to more strongly predict likelihood of an unplanned cesarean as opposed to patient knowledge, attitudes, and preferences [39]. Provider implicit bias towards racially minoritized patients may also deter patient-provider shared-decision making regarding cesarean birth [39]. Previous research has observed that positive attitudes towards vaginal birth did not vary by race and ethnicity but positive attitudes towards vaginal birth were associated with lower odds for cesarean only in birthing parents who were White and highly educated and had private insurance [40]. This research suggests that more attention may be given to White birthing parent preferences, thus impacting disparities in birth outcomes.

Strengths of this research included the use of a large contemporary dataset with a wide range of participating birth facilities across the Northwestern US. Using chart-abstracted data versus billing or administrative data allowed for more detailed analyses across socioeconomic variables and clinical indicators in pre-pregnancy, labor, and delivery. Unlike previously reported literature [5, 28], our data allowed for more detailed ethnic group analyses to explore the understudied issue of cesarean birth disparities by disaggregated Asian ethnic subgroups [8]. Limitations include missing data in race and ethnicity. Some facilities inconsistently captured Asian ethnic groups, instead using an “Asian unspecified” group and potentially diluting the effect given the heterogeneous outcomes of cesarean birth in the broader Asian group. Cultural factors that may contribute to disparities such as immigration status were not captured in the dataset. However, of the 15.5% of immigrants in Washington, 45% were born in Asia, so it is possible the Asian group may comprise a high degree of immigrants [41]. Variation in the quality of race and ethnicity data across birth facilities cannot be quantified. Our study did not control for COVID-19 pandemic factors that may have impacted cesarean trends. However, previous analyses of the OB COAP dataset did not identify any clear impact of COVID-19 on local cesarean rates [42]. Given that the makeup of the Asian population differs by regions of the USA, it may be challenging to generalize our findings to other locales.

Our study was the first, to our knowledge, to explore disparities in unplanned cesarean birth by disaggregated Asian groups using clinical data from the Northwestern US. This paper adds to the limited literature from other US regions demonstrating the larger “Asian” racial group’s masking of disparate cesarean outcomes [30–32]. Researchers and health systems should disaggregate the Asian race group given the wide variation in cesarean risk presented in this paper. According to the U.S. Department of Health and Human Services, best practices for disaggregating the Asian racial group include gathering data on detailed ethnicities that reflect diversity of the local community and pooling multiple months or years of data to address potentially small sample sizes [43]. Disaggregation allows for a more precise study of health outcomes and the development of culturally appropriate interventions. Understanding racial and ethnic disparities in cesarean outcomes within employer-sponsored health plans is critical for tailoring and promoting programming to higher risk groups. At the clinical level, without examining the disaggregated data, providers may be missing opportunities to mitigate lasting health impacts on birthing parents and babies. This understanding is essential for building trust and ensuring that health interventions are responsive to and respectful of cultural beliefs and practices. This paper is descriptive in nature, and more research is needed to further investigate causal pathways, areas for intervention, validate across other systems, and hear experiences from birthing parents and providers. However, as the US population becomes more diverse, being able to describe and address differences in outcomes by detailed race and ethnicity is essential for equity.

## Supplementary Information

Below is the link to the electronic supplementary material.

Supplementary file1 (PDF 210 KB)

## Data Availability

The data that support the findings of this study are from the Foundation for Health Care Quality's Obstetrical Care Outcomes Assessment Program (OB COAP), an ongoing perinatal quality improvement collaborative. Restrictions apply to the availability of these data, which were collected for quality improvement purposes and used under license for this study. Questions about availability of this data may be directed to fhcq@qualityhealth.org.
