# Supplementary material for: Racial and Ethnic Disparities in Low-Risk Unplanned Cesarean Birth: Disaggregating Asian Data
Source: J Racial Ethn Health Disparities. 2025 Mar 26;13(3):2094–102. doi: 10.1007/s40615-025-02401-0 (PMC13157395; doi:10.1007/s40615-025-02401-0)
Supplement: Supplementary file 1 — Supplementary file1 (PDF 210 KB) [file 40615_2025_2401_MOESM1_ESM.pdf]

Supplementary Information

Table 1: Patient Demographics by OMB 1997 Race Categories (Broad Race Groups)									
**Factor**	**AI/AN**	**Asian (Total)**	**B/AA**	**H/L**	**Multiracial**	**NH/PI**	**Other**	**White**	**Missing**
**Age** Mean (SD)	24.2 (5.55)	30.7 (4.12)	26.4 (5.72)	24.8 (5.57)	26.0 (5.62)	25.7 (5.21)	26.6 (5.34)	28.9 (5.38)	27.6 (5.56)
**BMI at Final Prenatal Visit** Median (IQR)	32.8 (28.8, 37.6)	28 (25.5, 30.9)	31.3 (27.8, 36.1)	31.6 (28.2, 36)	31.4 (27.9, 36.6)	34.7 (29.9, 39.7)	31.1 (27.6, 35.6)	30.8 (27.5, 35.3)	31.1 (27.6, 35.9)
**Baby Weight** (%)									
<2500g	10 (2.5)	348 (4.1)	91 (4.7)	111 (2.3)	10 (1.7)	12 (2.4)	25 (2.8)	395 (2.0)	57 (2.6)
2500-4000g	338 (83.9)	7878 (92.2)	1717 (89.6)	4499 (91.5)	552 (91.5)	447 (89.2)	825 (90.9)	17771 (88.3)	1952 (87.3)
>4000g	55 (13.6)	320 (3.7)	108 (5.6)	306 (6.2)	41 (6.8)	42 (8.4)	58 (6.4)	1966 (9.8)	226 (10.1)
**Insurance Type** (%)									
Government/Other	264 (65.5)	911 (10.7)	1145 (59.8)	2978 (60.6)	224 (37.1)	241 (48.1)	411 (45.3)	4633 (23.0)	985 (44.1)
Commercial	137 (34.0)	7557 (88.4)	765 (39.9)	1917 (39.0)	376 (62.4)	256 (51.1)	487 (53.6)	15324 (76.1)	1227 (54.9)
Missing/Unspecified	2 (0.5)	78 (0.9)	6 (0.3)	21 (0.4)	3 (0.5)	4 (0.8)	10 (1.1)	175 (0.9)	23 (1.0)
**Pre-pregnancy Hypertension** (%)	9 (2.2)	123 (1.4)	67 (3.5)	85 (1.7)	19 (3.2)	23 (4.6)	14 (1.5)	634 (3.1)	65 (2.9)
**Pre-pregnancy Diabetes** (%)	6 (1.5)	123 (1.4)	21 (1.1)	52 (1.1)	5 (0.8)	17 (3.4)	14 (1.5)	198 (1.0)	22 (1.0)
**DCI Quintile** (%)									
1 (Least distressed)	73 (18.1)	5786 (67.7)	486 (25.4)	1232 (25.1)	211 (35.0)	113 (22.6)	255 (28.1)	8933 (44.4)	630 (28.2)
2	84 (20.8)	1742 (20.4)	467 (24.4)	951 (19.3)	170 (28.2)	128 (25.5)	259 (28.5)	6009 (29.8)	764 (34.2)
3	32 (7.9)	668 (7.8)	466 (24.3)	702 (14.3)	67 (11.1)	101 (20.2)	191 (21.0)	2273 (11.3)	304 (13.6)
4	116 (28.8)	262 (3.1)	373 (19.5)	1473 (30.0)	104 (17.2)	112 (22.4)	143 (15.7)	2097 (10.4)	387 (17.3)
5 (Most distressed)	84 (20.8)	51 (0.6)	108 (5.6)	510 (10.4)	46 (7.6)	39 (7.8)	54 (5.9)	574 (2.9)	111 (5.0)
Missing	14 (3.5)	37 (0.4)	16 (0.8)	48 (1.0)	5 (0.8)	8 (1.6)	6 (0.7)	246 (1.2)	39 (1.7)
**Preeclampsia/Gestational Hypertension** (%)	61 (15.1)	745 (8.7)	312 (16.3)	618 (12.6)	65 (10.8)	83 (16.6)	125 (13.8)	3221 (16.0)	357 (16.0)
**Gestational Diabetes** (%)	24 (6.0)	1457 (17.0)	122 (6.4)	376 (7.6)	48 (8.0)	49 (9.8)	76 (8.4)	1463 (7.3)	183 (8.2)
**Induced** (%)	156 (38.7)	3219 (37.7)	888 (46.3)	1843 (37.5)	230 (38.1)	193 (38.5)	348 (38.3)	8935 (44.4)	1005 (45.0)
**Neonatal Level of Care** (%)									
1	43 (10.7)	105 (1.2)	69 (3.6)	594 (12.1)	4 (0.7)	22 (4.4)	32 (3.5)	1674 (8.3)	209 (9.4)
2	58 (14.4)	658 (7.7)	352 (18.4)	688 (14.0)	168 (27.9)	125 (25.0)	232 (25.6)	5181 (25.7)	1052 (47.1)
3 & 4	302 (74.9)	7783 (91.1)	1495 (78.0)	3634 (73.9)	431 (71.5)	354 (70.7)	644 (70.9)	13277(65.9)	974 (43.6)
**Absent/Minimal Prenatal Care** (%)	37 (9.2)	96 (1.1)	157 (8.2)	203 (4.1)	23 (3.8)	48 (9.6)	45 (5.0)	388 (1.9)	99 (4.4)

AI/AN – American Indian/Alaska Native; B/AA – Black/African America; H/L – Hispanic/Latinx; NH/PI – Native Hawaiian/Pacific Islander

Submission Details:

Title: Racial and ethnic disparities in low-risk unplanned cesarean birth: Disaggregating Asian Data

Journal: Journal of Racial and Ethnic Health Disparities

Author information: Ms. Sydney M SPENCER, MPH[1,2]; Ms. Amy A LAURENT, MSPH[1]; Dr. Vivienne L SOUTER, MD,[3] Dr. Ian S PAINTER, PhD[3]; Dr. Colleen M DALY, PhD[1]

Author Affiliation: [1] Microsoft Corporation, Redmond, WA, USA**;** [2] University of Washington, Department of Health Systems & Population Health, Seattle, WA, USA; [3] Foundation for Healthcare Quality, Seattle, WA, USA

Corresponding author/email address: Ms. Sydney M SPENCER, MPH sspenc4@uw.edu
